# Dr. Miller in Reply to Dr. Patterson

**Published:** 1803-04-01

**Authors:** W. Patterson

**Affiliations:** Londonderry


### To Dr. BRADLEY.

DEAR SIR,

SINCE I wrote to you on the 19th instant, I have been favoured with a communication from Dr. Miller of New-York, stating the causes which prevented the appearance of my "*Reply*" in the Repository, and of which, in justice to Dr. M——'s candour and liberality, I shall make an abstract for insertion in the Medical Journal.

"After learning the real state of the case," says Dr. M——, "I am confident you will be less disposed to admit the suspicion of any improper motive in my conduct or in that of my colleague, Dr. Mitchill."

With respect to one point, namely, the interchange of letters, the interruption which happened on the part of Dr. Miller, I am truly sorry to find, was occasioned by the invasion of phthisical symptoms, from which he did not recover for several months, at which period no vessels were up for Londonderry, and none afterwards offered until the present season.

"As to your *Reply*," continues Dr. Miller, to the Strictures which appeared in the Medical Repository, on the opinions of yourself and Dr. Drennan, *it never came to hand.* If it had ever reached my hand, it assuredly would have found a place in the Medical Repository; for I never will be the advocate of opinions which require to be defended by the suppression of fair argument urged in opposition. If you will favour me with another copy, the Reply shall still be inserted. I suspect that others too of your communications to me have failed, from the vessels to which they were committed arriving at some distant part of the United States, instead of New York.

"I much fear that you and I cannot agree on the origin of pestilential diseases. But it does not follow from thence, that we cannot entertain the most sincere and candid esteem for one another, or that there is the least difficulty in reciprocating every obliging and kindly office. To cultivate the most perfect harmony, good understanding, and and liberal intercourse with you, is my warm and unaffected desire. Let us not then suffer any improper suggestions, arising from any quarter, especially from suspicious ones, to make any hasty impressions on our minds, until the full evidence of facts, and indisputable facts, is set before us."

Most sincerely do I regret, that an unsuspected mischance should have given me the least reason to harbour for a moment the smallest idea adverse to the candour of a correspondent actuated by such generous and philosophical sentiments. As the circumstances, therefore, which influenced my reflections relative to the Editors of the Medical Repository, are before the public, it is incumbent on me to give equal publicity to my revocation of whatsoever has appeared from me unfavourable to the liberality of their print. And, I trust, that every reader of my *Reply*, in the Medical Journal, will do me the justice to allow, that I have not, in any one part of it, trespassed against the principles of fair and temperate discussion.

My esteemed correspondent, Dr. Miller, declares himself a sincere and ardent lover of peace, the value of which he "sees more and more every day; insomuch that, for the sake of securing it, he is disposed to yield trifles, and even some things of more consequence. I can assure my friend, Dr. M.——, as I have more than once already done, that he cannot be more conscientiously devoted to pacific terms of disquisition than I am, as being the surest avenue to truth and solid knowledge; and I further assure him, that I am still governed by the sentiment which I before expressed to him:

'Nobis magis cordi est pacificus veritatis ingressus, quam qui pugnax est, 'viamque sibi per contentiones, et lites sternat.'

Perhaps some of the readers of the Medical Journal may be able to give an instructive answer to the following question, asked by Dr. Miller:—"Can you direct me to any source of full and satisfactory information concerning the best method of discharging from cities the filth of animal and vegetable kinds, constantly accumulating in them, and especially the filth of privies? We are engaged in endeavouring to make some improvements of this description in New-York, and wish to be instructed and assisted by the experience and wisdom of older cities."

Give me leave to second this wish of Dr. Miller, and to interest on the occasion your ingenious and philanthropic correspondents, whose contributions will very much gratify,

Your's, &c.

*Londonderry, Feb. 26, 1803.* W. PATTERSON.

### Extract *of a* Letter *dated December* 1*st*, 1802, *from a Physician in Philadelphia, to* Dr. Patterson *in Londonderry.*

"I HAVE now perused the abstract of my letter, with your notes, published in the Medical and Physical Journal, No. xxxviii; and am perfectly satisfied with what you have done, as I believe your motive was a laudable one. However, I have committed some mistakes in that letter, with respect to the doctrines advanced by Dr. Latham Mitchill, of New York; and I therefore request, that you will do me the favour of transcribing the following correction, for insertion in the Medical and Physical Journal, as it would give me great concern to be thought capable of designedly misrepresenting any opinion in what manner soever it may be founded.

In stating the objections of Mr. Lee*, a medical student of this city, to Dr. Mitchill's doctrine, I have inadvertently omitted a paragraph, which gives those objections the appearance of inconsistency, and I have incorrectly said, Dr. Mitchill "denominates the *nitrous acid*, in a gaseous state, *gaseous oxyd of septon*;"† whereas, I should have said, Dr. Mitchill supposes that a substance, which he calls the gaseous oxyd of septon, and which is denominated by other chemists dephlogisticated nitrous air, is one of the products of putrefaction, and is the cause of pestilential fevers, &c. But the dephlogisticated nitrous air differs so much from common nitrous air, in consequence of the different proportions of the bases of azote and oxygen, of which, in conjunction with caloric, it is composed, as to produce exhilarating and salutary effects, instead of pestilential and destructive ones. I might also have added, that I have the authority of Professor Woodhouse to say, there are no proofs that such a substance as that called the gaseous oxyd of septon is ever formed by, or in consequence of, the process of putrefaction, though the nitrous or septic acid is a plentiful product of that process.

Dr. Strother,‡ who early in the last century, occupied much of his time in abstract speculations instead of experimental

* This ingenious young gentleman, who bid fair to be an ornament to science, died last February of phthisis.

† Medical and Physical Journal, vol. vii. p. 317.

‡ Strother on Small-Pox and Plague, London, printed A. D. 1721.

perimental inquiries, imagined that, because strong acids produced vesications and corrosion of the skin when externally applied, they occasionally produced the small-pox and the plague, in certain situations where the air is highly impregnated with them, particularly by that acid which produces nitre on brick walls and in cellars. How far this doctrine agrees with Dr. Mitchill's relative to the cause of the malignant fever and the dysentery, I leave to the sagacity of Dr. Mitchill to decide.

For other objections to the doctrine of the New-York professor, I beg leave to refer to the Monthly Review, for 1797, vol. XXIII. p. 553, and to Med. Repos. vol. I. p. 246."

